# Topical timolol maleate 0.5% after fractional carbon dioxide laser versus fractional carbon dioxide laser alone in treatment of acne scars: split face comparative study

**DOI:** 10.1038/s41598-023-36398-5

**Published:** 2023-06-09

**Authors:** Aya Reda Mohamed Hawwas, Hassan Abou Khodair Mohamed, Osama Magdy Elshahat Sayedahmed, Mohamed L. Elsaie

**Affiliations:** 1 Department of Dermatology, Venereology and Andrology, Damietta Faculty of Medicine, Al-Azhar University, Damietta, Egypt; 2 Department of Dermatology, Medical and Clinical Research Institute, National Research Centre, Dokki, Egypt

**Keywords:** Diseases, Medical research

## Abstract

Acne is a common inflammatory condition that mostly involves the face, chest and back. A number of different modalities had been employed for treating scars of which laser remains to be a pivotal choice. We aimed to compare the efficacy of topical timolol maleate 0.5% after fractional CO2 (AFCO2) laser versus fractional CO2 Laser alone for treatment of atrophic acne scars. A split-face comparative clinical experiment on 30 cases of atrophic post-acne scars that were treated on one side with ablative fractional CO2 laser followed by timolol application while with only ablative fractional CO2 laser on the other side. Following treatment, both sides demonstrated significant improvement with the laser + timolol treated side showing better improvement; yet not significantly higher than the laser only treated side. In conclusion, both topical timolol maleate 0.5% after fractional CO2 laser and fractional CO2 laser may achieve comparable significant improvement. The good safety profile, easy accessibility, low cost, and non-invasive nature merits the use of timolol in acne scars pending verification by larger sample reproduced and controlled trials.

## Introduction

Acne is a common inflammatory condition that mostly involves the face, chest and back with many patients experiencing some degree of scarring, the severity of which correlates to acne grade^1^.

Acne scars are more common on the face than anywhere else on the body and can affect up to 70% of acne patients^2^. Recent reports suggested that the severity of acne, time since its onset and starting treatment, family history as well as number of relapses are key factors related to scar formation^3^. Atrophic scars are the commonest form in acne patients and can be subdivided into ice pick, boxcar or rolling^1^.

Acne scars can negatively impact quality of life, impair functionality and can inflict low self esteem on patients^4^. Different modalities of treatment have been used to improve the appearance of acne scars with variable clinical results, as surgical techniques (subcision, punch graft, or punch excision), resurfacing techniques (chemical peeling or ablative laser resurfacing) that has long downtime duration despite of their good improvement effects. Over the last years, other relatively new therapeutic modalities have been introduced such as microneedling, energy based devices (EBDs) and platelet rich plasma (PRP). Despite of all modalities employed, yet scar treatment remains to be a challenge of which laser treatments remains to be a pivotal choice^5^.

Timolol (TM) is a beta adrenergic receptor blocker that had been employed recently in treating a number of skin conditions. The role of TM in skin wound healing was identified through its ability to induce fibroblast proliferation and regulation collagen remodeling in extracellular matrix^6^.

Taking into consideration the potential role of TM in wound healing and collagen remodeling, this study aimed to compare the efficacy of topical timolol maleate 0.5% after fractional CO2 (AFCO2) laser versus fractional CO2 Laser alone for treatment of atrophic acne scars.

## Patients and methods

This comparative clinical trial split face approach was carried out on 30 cases of atrophic post-acne non erythematous scars that were diagnosed clinically on the basis of typical appearance of skin lesions. Patients were recruited from the Dermatology and andrology outpatient clinic at Damietta Faculty of Medicine, Al-Azhar University from January 2022 to May 2022. This study protocol was reviewed and approved by ethics committee on human research by Al Azhar Damietta faculty of medicine (No. IRB00012367-19-07-000). All methods were performed in accordance with the relevant guidelines and regulations. Subjects were briefed about the procedural treatment and the expected consequences and signed informed consents were received for participation and publishing of obtained photos.

Facial acne scars and a minimum age of 18 years were both required for inclusion. Pregnant or nursing women, those suffering from aggressive inflammatory acne, and active infection in the treatment area (e.g., verrucae and herpes simplex) were excluded. Subjects with known systemic disease (hypertension, diabetes or bleeding tendency), on anticoagulant therapy as well as history of keloidal tendency were excluded from the study (supplementary material).

### Study procedures

Thirty cases (30) of bilateral atrophic post-acne scars assessed clinically were recruited from the university hospital's outpatient clinic. All patients were subjected to full history taking, complete general examination, scar assessment for the type of scar (ice pick, boxcar or rolling) and baseline photographs were obtained using a Nikon D5300 camera. All methods were performed in accordance with the relevant guidelines and regulations.

Topical anaesthetic cream containing an eutectic mixture of topical tetracaine and lignocaine in a cream base (Tetralid^R^ cream) were applied for 1 h on the treatment area to achieve a satisfactory anaesthetic effect. After satisfactory anaesthesia was achieved; fractional CO2 laser (*SmartXide* DOT; *DEKA*, Calenzano, *Italy*) treatment was done to each atrophic scar present. Fluence ranging from 2.8 to 3.5 J/cm^2^ was used at power of 15–20 W and a dwell time of 0.5 ms, thus providing about 25–30 mJ of energy and density of 13.5%. A double pass was used over each scar along with its margins. Each morphological type of scar was treated in a similar manner and the patient was advised skin cooling with ice-packs for 5–10 min after the procedure to take care of post-treatment erythema, oedema and burning sensation.

Following laser treatment, subjects applied 10–15 drops of TM 0.5% solution (Timolol^R^, Epico pharma, Egypt) to the right side of their cheeks only. The patients were instructed to prevent sun exposure for the next 4–5 days and instructed to apply 10–15 drops of TM solution 0.5% on the right side of the face, and continued to apply the solution in the very same manner twice daily for 7 consecutive days. They were also advised to apply (in-house prepared hydrophilic cream) and sunscreen on both sides of the face. No other topical solutions or preparations were used during the study period. Laser procedures were repeated every 5 weeks and a total of 3–4 sessions were performed for each patient. The laser parameters were kept identical at each visit and if the patient satisfied with the results obtained after the 3rd session, the 4th session not performed.

Digital photographs were taken using identical angles and face position settings at every visit and at the final follow up visit 8 weeks after the last laser session.

### Outcome evaluation

Acne scar assessment scale (ASAS) assessing the severity of scars and ranging from 0 (Clear) to 4 (Severe) as well as Scar quartile grading scale (SQGS).degree of improvement Grade 0: no improvement, Grade 1: Less than 25% improvement, Grade 2: 26–50% improvement, Grade 3: 51–75% improvement and Grade 4: More than 75% improvement were evaluated by pre and post procedural digital photographs assessed by an independent blinded dermatologist and results were graded on the basis of the percentage improvement^7^.

### Patient satisfaction

Subjective levels of satisfaction were questioned at the exit laser visit in comparison to the pre-treatment state on both sides and were graded as excellent, good, fair or poor.

### Statistical analysis of data

The data was gathered, edited, coded, and put into IBM SPSS (Statistical Package for Social Science) version 23. The quantitative data distribution was judged to be parametric, mean and standard deviations were provided, but data with non-parametric distributions were presented as median with inter-quartile ranges (IQR). To indicate qualitative features, numbers and percentages were also used. The confidence interval was set at 95%, while the acceptable margin of error was set at 5%. As a result, the *p*-value was deemed significant if less than < 0.05.

### Study approval statement

This study protocol was reviewed and approved by ethics committee on human research by Al Azhar Damietta faculty of medicine (No. IRB00012367-19-07-000). All methods were performed in accordance with the relevant guidelines and regulations.

### Consent to participate statement

Written informed consents were received from participants upon explanation of the study. Consent for publication were obtained from the participants for publishing the images in the manuscript.

## Results

In the present study, the mean age was 25.63 ± 3.76 years; there were 11 (36.7%) male and 19 (63.3%) female subjects. The mean duration of acne scars was 7.3 ± 3.26 years. Seven (7) subjects complained of ice pick scars, fourteen (14) had ice pick scars, six (6) had boxcar scars while only three (3) subjects presented with mixed scars.

**Figure 1:**
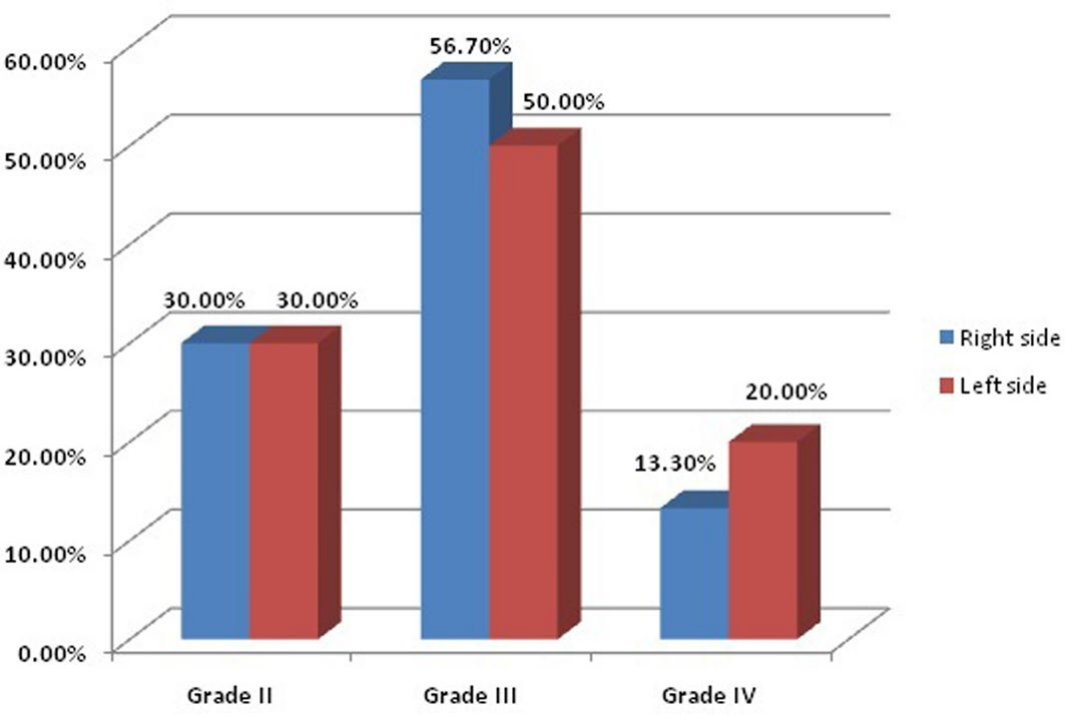
Acne Scar Assessment Scale before treatment of the studied cases.

**Figure 2:**
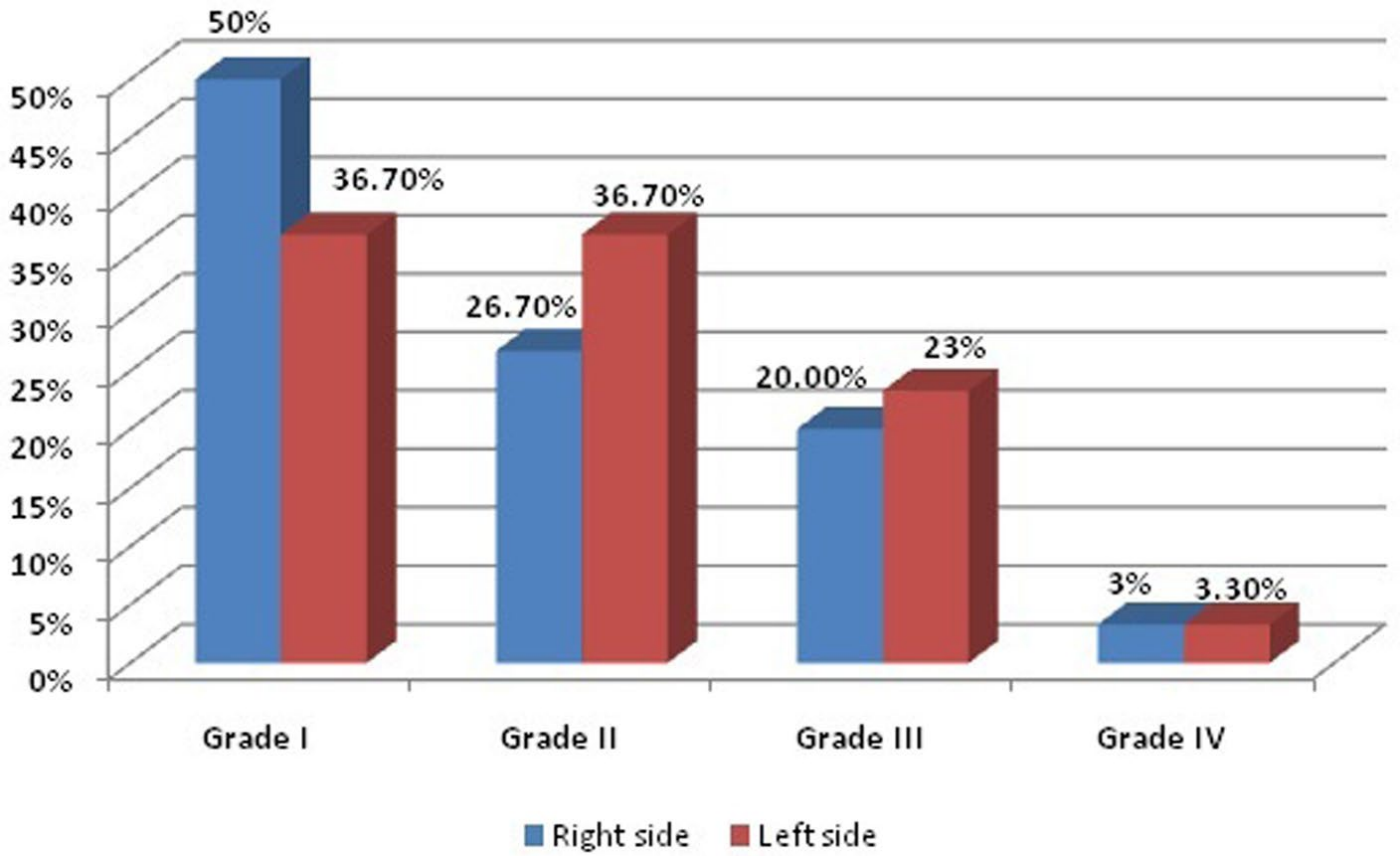
Acne Scar Assessment Scale after treatment of the studied cases.

Before management, regarding acne scar assessment scale (ASAS) scar grades ranged from 2–4 with no significant difference on both treated sites. Following treatment, both sides demonstrated significant improvement with the AFCO2 + TM 0.5% treated side ( right side) showing better improvement; yet not significantly higher than the AFCO2 only treated side ( left side) (Table 1; Figs. 1–2).

**Table 1:** Acne Scar Assessment Scale before and after treatment of the studied cases regarding right and left side.

	Variables	Before		After		Test significance	*P* value
		No	%	No	%		
Grading in right side							
AFCO2 + TM	Grade I	0	0.0%	15	50.0%	20.35	< 0.001*
	Grade II	9	30.0%	8	26.7%		
	Grade III	17	56.7%	6	20.0%		
	Grade IV	4	13.3%	1	3.3%		
Grading in left side							
AFCO2	Grade I	0	0.0%	11	36.7%	15.98	0.001*
	Grade II	9	30.0%	11	36.7%		
	Grade III	15	50.0%	7	23.3%		
	Grade IV	6	20.0%	1	3.3%		

**Fr**: **Friedman test**, Sig. betdifferent treatments was done using** Post Hoc Test** (**Dunn's).**

p: *p* value for association between different treatments*: Statistically significant at p ≤ 0.05.

AFCO2 + TM : ablative fractional carbon dioxide laser and Timolol 5% solution.

AFCO2: ablative fractional carbon dioxide laser.

**Figure 3:**
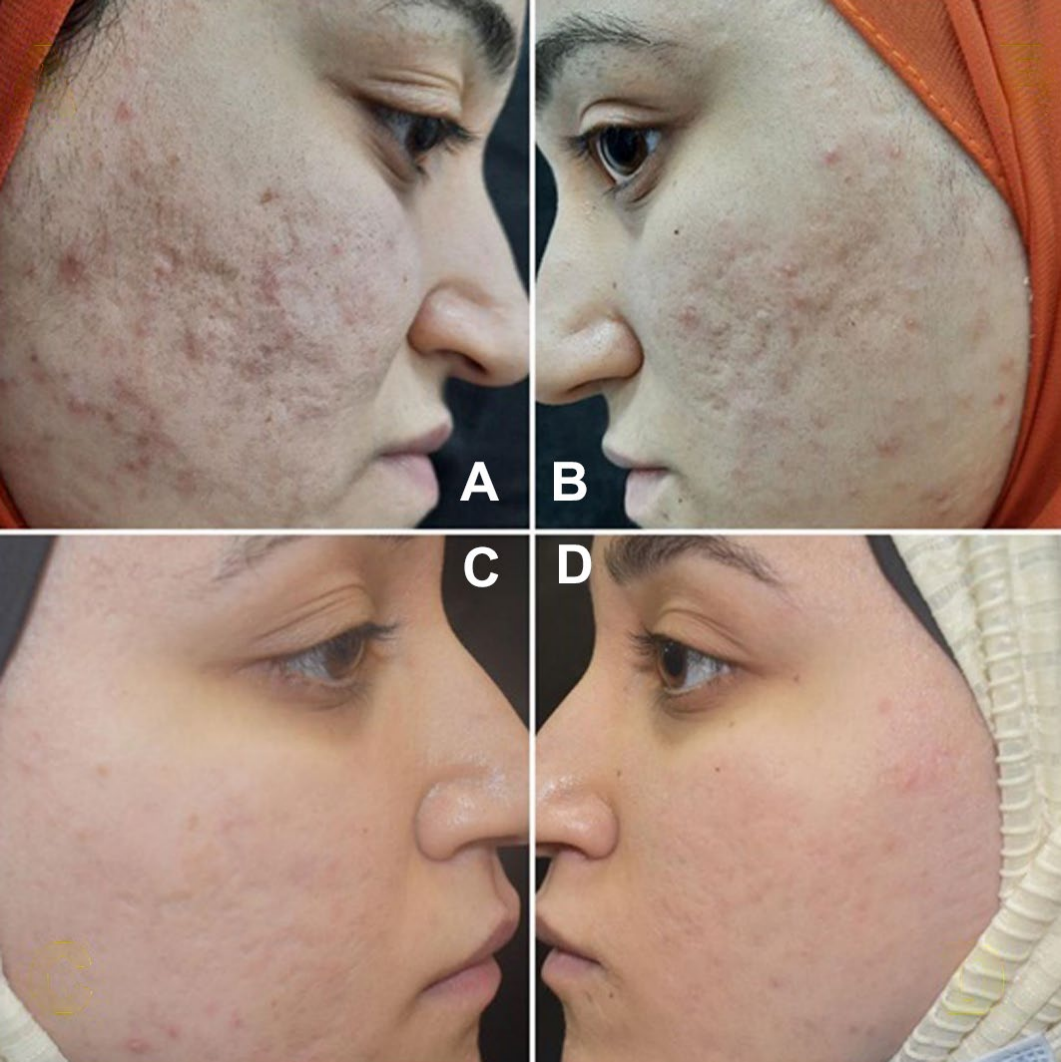
Clinical images of a 22 years old female (**A**) right side at baseline; (**B**) left side at baseline; (**C**) laser + timolol treated side 8 weeks after last session and (**D**) laser only treated side, 8 weeks after last laser treatment.

**Figure 4:**
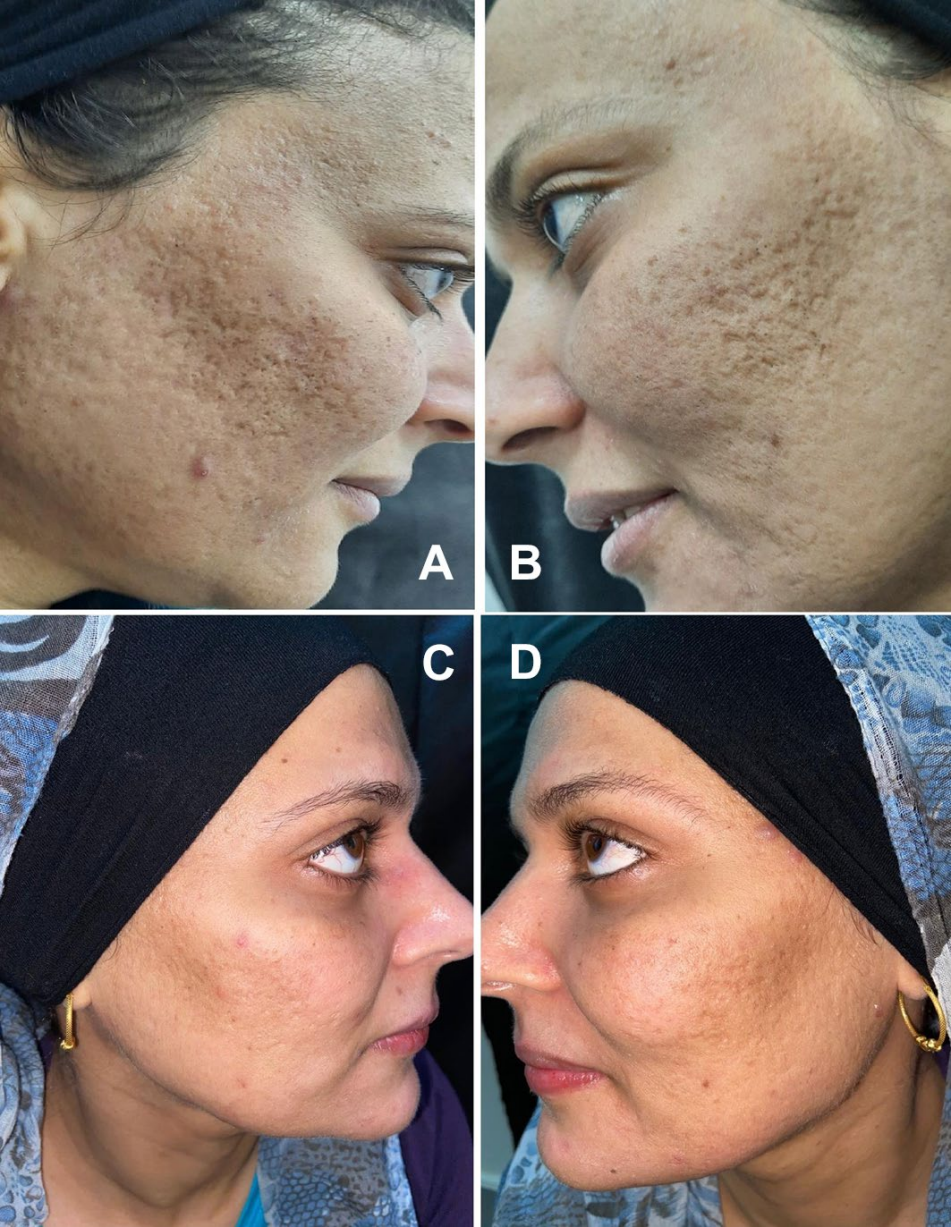
Clinical images of a 32 years old female (**A**) right side at baseline; (**B**) left side at baseline; (**C**) laser + timolol treated side 8 weeks after last session and (**D**) laser only treated side, 8 weeks after last laser treatment.

Another assessment parameter used was SQGS. On the right half (AFCO2 + TM 0.5%), 4 patients showed more than > 75% improvement, 9 patients showed 51–75% improvement, 11 patients showed 26–50% improvement, 5 patients showed < 25% improvement and only 1 patient reported no improvement whereas on the left side ( AFCO2) > 75% improvement was reported by 1 patient, 51–75% improvement in 5 patients, 26–50% improvement in 11 patients, < 25% improvement in 10 patients and no improvement reported by 3 patients. The overall improvement reported was significant in both sides (p < 0.05); yet comparable in both modalities used (p = 0.23) (Table 2; Figs. 3–4).

**Table 2:** Scar quartile grading scale (SQGS).degreeof improvement of the studied cases regarding 0.5% TM after AFCO2 and AFCO2.

Variables	Right side (AFCO2 + TM)	Left side (AFCO2)	Test significance	*P* value
Grade 0	1 (3.3%)	3 (10.0%)	5.61	0.23
Grade I	5 (16.7%)	10 (33.3%)		
Grade II	11 (36.7%)	11 (36.7%)		
Grade III	9 (30.0%)	5 (16.7%)		
Grade IV	4 (13.3%)	1 (3.3%)		

**Fr**: **Friedman test**, Sig. betdifferent treatments was done using** Post Hoc Test** (**Dunn's).**

p: *p* value for association between different treatments*: Statistically significant at p ≤ 0.05.

AFCO2 + TM : ablative fractional carbon dioxide laser and Timolol 5% solution.

AFCO2: ablative fractional carbon dioxide laser.

Patients reported a significant satisfaction (P < 0.05) on both sides yet comparable on both sides (p = 0.12). On the AFCO2 + TM 0.5% (right) treated side, 8 patients reported excellent levels of satisfaction and 15 showed good satisfaction; while on the AFCO2 only (left) treated side, 3 patients showed excellent satisfaction and 12 reported good levels of satisfaction (Table 3).

**Table 3:** patient satisfaction of the studied cases regarding right and left side.

Variables	Right side (AFCO2 + TM)	Left side (AFCO2)	Test significance	*P* value
Poor	1 (3.3%)	3 (10.0%)	5.61	0.12
Fair	6 (20.0%)	12 (40.0%)		
Good	15 (50.0%)	12 (40.0%)		
Excellent	8 (26.7%)	3 (10.0%)		

**Fr**: **Friedman test**, Sig. betdifferent treatments was done using** Post Hoc Test** (**Dunn's).**

p: *p* value for association between different treatments*: Statistically significant at p ≤ 0.05.

AFCO2 + TM : ablative fractional carbon dioxide laser and Timolol 5% solution.

AFCO2: ablative fractional carbon dioxide laser.

On the right side (AFCO2 + TM 0.5%), 2 (6.7%) patients experienced erythema, 1 subject (3.3%) reported edema, 1(3.3%) subject showed infection, and only 1 subject (3.3%) suffered hyperpigmentation. On the left side (AFCO2), 4 (13.3%) patients experienced erythema, 2 subjects (6.7%) reported edema, and 3 subjects (10.0%) suffered hyperpigmentation. Side effects were comparable and insignificantly different in both sides of the face (p = 0.83) (Table 4).

**Table 4:** Side effect after management of the studied cases 0.5% TM after AFCO2 and AFCO2.

Variables	Right side (AFCO2 + TM)	Left side (AFCO2)	Test significance	*P* value
Erythema	2 (6.7%)	4 (13.3%)	1.45	0.83
Edema	1 (3.3%)	2 (6.7%)		
Infection	1 (3.3%)	0 (0.0%)		
Hyperpigmentation	1 (3.3%)	3 (10.0%)		
Pain	3 (10.0%)	2 (6.7%)		

**Fr**: **Friedman test**, Sig. betdifferent treatments was done using** Post Hoc Test** (**Dunn's).**

p: *p* value for association between different treatments*: Statistically significant at p ≤ 0.05.

AFCO2 + TM : ablative fractional carbon dioxide laser and Timolol 5% solution.

AFCO2: ablative fractional carbon dioxide laser.

## Discussion

Atrophic acne scars are a challenging condition to treat and AFCO2 had been one of the most efficacious modalities used to date^8^. Facilitation of drug delivery has been utilized and implemented in a range of skin conditions following ablative laser treatments. Photothermolysis and the formation of microthermal zones (MTZs) facilitate the delivery of high molecular weight molecules into the stratrum cornerum and skin layers through the preformed channels^9^.

The present study provided an insight into the safety and efficacy of laser assisted delivery of topical timolol 0.5% solution in subjects suffering of acne scars. The results showed a favorable better outcome on the timolol treated site though comparable and non significant to laser only treated site.

Similar to our results a recent double blinded placebo controlled trial on 25 patients complaining of acne scars demonstrated that application of topical 0.5% TM twice daily on one side of the face improves the skin-barrier function and promoted re-epithelialization after laser procedures when compared to the placebo controlled side where normal saline was only applied^10^.

Beta adrenergic receptors are widely present in the human body and their Beta 2 adrenergic receptor subgroup expression in human keratinocytes was first reported years ago^11^. Timolol is a beta adrenergic blocker that was reported to stimulate wound healing by facilitating keratinocyte migration by increasing phosphorylation of extracellular signal related kinases (ERKs)^12^. Moreover ERKs were shown to regulate fibroblast function and help in collagen remodeling of the extracellular matrix^13,14^.

Acute wound treatment using timolol following surgical excision of skin cancer was investigated in six (6) patients and showed that the use of topical timolol on acute wounds improved the aesthetic results twice more in the wound site^15^.

Another report, showed an increase in the rate of wound healing caused by destructive CO_2_ laser after using topical timolol. Treatment sites ablated by laser for which topical timolol 0.5% was applied demonstrated less inflammation and a significantly lower transepidermal water loss (TEWL) than ablated areas where no timolol was applied^16^.

Moreover; timolol was found to induces apoptosis, inhibit angiogenic factors, such as vascular endothelial growth factor (VEGF), and inflammatory mediators, such as matrix metalloproteinase (MMP)-2, MMP-9, and interleukin (IL)-6. Such properties had demonstrated its potential usefulness for treating acne and rosacea^17^.

Application of 0.5% timolol after TCA-CROSS in patients complaining of acne scars was found to slightly decrease scar severity and result in a significant reduction of post-inflammatory hyperpigmentation (PIH) duration^18^.

No significant side effects were reported in the present study which was supported by other reports in literature demonstrating a high safety profile of timolol^19^. Our study's limitations include a small sample size, short follow up period as well as failure of plasma or serum assessment of timolol levels to establish any systemic absorption potential.

## Conclusion

To the best of our knowledge, no studies have reported the comparing of the efficacy of topical timolol maleate 0.5% after fractional CO2 laser versus fractional CO2 laser for treatment of atrophic acne scars. The results showed a comparable non significant outcome on the timolol treated site when compared to the laser only treated site. Timolol did not add a significant better outcome to the laser treatment pending verification by larger sample reproduced and controlled trials.

## Supplementary Information

Supplementary Information.

## Data Availability

The data that support the findings of this study are available from the corresponding author upon reasonable request.
