# Supplementary material for: Topical timolol maleate 0.5% after fractional carbon dioxide laser versus fractional carbon dioxide laser alone in treatment of acne scars: split face comparative study
Source: Sci Rep. 2023 Jun 9;13:9402. doi: 10.1038/s41598-023-36398-5 (PMC10256714; doi:10.1038/s41598-023-36398-5)
Supplement: Supplementary file 1 — Supplementary Information. [file 41598_2023_36398_MOESM1_ESM.doc]

**Sample size calculation:**

Using the following calculation, the study's sample size was determined to be (30) individuals at a 5% level of significance and 90 percent power (Daniel, 1999)

Z2 * P* (1-P)

N=

d2

Where;

Z= 1.96 for 95% confidence level.

p = the expected efficacy of Topical Timolol Maleate 0.5% After Fractional Carbon-Dioxide Laser Versus fractional carbon dioxide laser alone (81%).

d = precision (Margin of error) = 0.03
